# Meta-Analysis of Personality Traits in Alzheimer’s Disease: A Comparison with Healthy Subjects

**DOI:** 10.3233/JAD-170901

**Published:** 2018-02-20

**Authors:** Alfonsina D’Iorio, Federica Garramone, Fausta Piscopo, Chiara Baiano, Simona Raimo, Gabriella Santangelo

**Affiliations:** Department of Psychology, University of Campania “Luigi Vanvitelli”, Caserta, Italy

**Keywords:** Alzheimer’s disease, dementia, factor risk, neuroticism, personality

## Abstract

**Background:**

The role of specific personality traits as factor risks of Alzheimer’s disease (AD) has been consistently found, whereas personality traits specifically related to AD (after the diagnosis) have not been outlined yet.

**Objective:**

A meta-analysis of published studies was performed to determine whether AD patients have a distinctive personality trait profile compared to healthy subjects (HC), similar to or different from a premorbid personality profile consistently reported in previous studies.

**Methods:**

A systematic literature search was performed using PsycInfo (PROQUEST), PubMed, and Scopus. The meta-analysis pooled results from primary studies using Hedges’ g unbiased approach.

**Results:**

The meta-analysis included 10 primary studies and revealed that, when the personality was evaluated by informant-rated measures, AD patients had significantly higher levels of Neuroticism, lower levels of Openness, Agreeableness, Conscientiousness, and Extraversion than HCs. When the personality was evaluated by self-rated measures, the results obtained from informants were confirmed for Neuroticism, Openness, and Extraversion but not for Agreeableness and Conscientiousness where AD patients and HCs achieved similar scores.

**Conclusions:**

The meta-analysis revealed that high Neuroticism and low Openness and Extraversion are distinctive personality traits significantly associated with a diagnosis of AD when evaluated both self-rated and informant-rated measures. This personality trait profile is similar to premorbid one, which contributes to development of AD over time. Therefore, our findings indirectly support the idea of specific premorbid personality traits as harbingers of AD.

## INTRODUCTION

Personality is defined as a set of psychological qualities contributing to distinctive types of feelings, ways of thinking and behaviors [1]. Some authors proposed that individuals are characterized by certain personality traits that partially determine their behaviors.

Personality changes may reflect structural and functional alterations produced by the progressive neurodegenerative processes occurring in neurological diseases such as in Alzheimer’s disease (AD). Several patterns of personality changes associated with AD are possible. Some authors proposed that a premorbid personality might determine personality changes with caricature or exaggeration of the original personality (as previously reported [2]). Others reported that the onset of AD might lead to a specific disease profile labeled as “Alzheimer personality” [3, 4], which is why patients with AD might show a similar behavioral profile. Others proposed that the personality changes in a stereotypic way [4, 5] and so AD patients show a reduction or an increase in personality characteristics while maintaining individual variability since AD patients showed personality changes but those who scored the highest level on a particular premorbid trait remained on the highest trait even after AD onset [4–6]. Finally, personality changes in AD might occur at random, without a pattern or consistency [2]. Some systematic reviews and meta-analysis focused on personality traits defined in terms of the Five-Factor Models (FFM, i.e., Neuroticism, Extraversion, Openness to experience, Agreeableness, Conscientiousness) [7] and their changes over time (before and after the diagnosis) or on identification of personality traits associated with a high risk of developing dementia. In a review, Robins Wahlin et al. [8], investigated the change in each of the five traits over time in AD and revealed that Conscientiousness and Neuroticism are the personality traits that exhibit the biggest changes in dementia and therefore they might be useful as early markers of dementia. Some recent meta-analysis supported the association between Neuroticism and Conscientiousness with cognitive decline; in particular, higher levels of Neuroticism were associated with an increased risk of dementia and higher levels of Conscientiousness were protective against its incidence [9]. A recent meta-analysis, which included a small number of primary prospective studies (*n* = 5), confirmed this [10], and added that a high level of Openness and Agreeableness was associated with a lower risk of AD, while no significant association with Extraversion was found. It is noteworthy to underline that whereas the role of specific personality traits as factor risks of AD has been consistently found, a personality profile specifically related to AD (after the diagnosis) has not yet been outlined. Several studies have explored personality profile of AD patients compared to healthy subjects (HC), and found more consistent results for Neuroticism and mixed findings for Extraversion, Agreeableness, Conscientiousness, and Openness [4, 11–16]. Therefore, in our study, we conducted a meta-analysis to determine whether patients with a clinical diagnosis of AD have a distinctive personality trait profile compared to healthy subjects. Based on previous research, we would explore whether a personality trait profile associated with AD is similar to or different from a premorbid personality profile consistently reported in previous studies (i.e., high Neuroticism and low Conscientiousness). Moreover, we investigated whether demographic or clinical aspects and type of instruments to assess personality traits would have an influence on the meta-analytic outcomes.

The current meta-analysis might shed light on a stereotypic personality profile in AD patients whose early identification may be helpful in formulating a clinical diagnosis; it can also aid in care management, anticipating difficult issues in the progression and treatment of the disease. Moreover, at a broader level, information on personality traits can be useful to clinicians to modulate their interactions with AD patients. Finally, the present study could help researchers identify personality traits distinctive for older adults with dementia in order to build and design socially intelligent robots adapting to their behaviors and needs, in a Social Assistive Robotics (SAR) [17] perspective. SAR promotes the goal to provide assistance to human users, but it specifies that the assistance is through social interaction between the robot and human user. Therefore, the robot’s goal is to create close and effective interaction with a human user for the purpose of giving assistance and achieving measurable progress in convalescence, rehabilitation, and learning [18].

## MATERIALS AND METHODS

### Search strategy and study eligibility criteria

A systematic literature search was performed on 10 June 2017 using PsycInfo (PROQUEST), PubMed, Scopus, with the following search terms: “personality” or “temperament” or “neurot*” or “negative emotionality” or “extraversion” or “introversion” or “openness to experience” or “cognitive rigidity” or “rigidity” or “agreeableness” or “conscientiousness” or “impulsiv*” or “novelty seeking” or “harm avoidance” or “reward dependence” or “persistence” and “dementia”. This search was supplemented by hand searches of reference lists cited in the original and review articles. Studies were included in the meta-analysis if they: 1) were published in peer-reviewed journals in English; 2) were published from 1960 to March 2017; 3) compared patients with AD to HC on personality traits related to the FFM; and 5)reported statistical results about comparisons on personality traits between AD and HC.

We excluded conference proceedings, letters to the editor, theses, animals and single case studies, commentaries, and studies investigating patients with non-Alzheimer dementias (e.g., frontotemporal dementia, vascular dementia). Where the same data were presented in more than one publication, we used the primary (first) publication.

All aspects of study selection, extraction, and assessment were performed by reviewers working independently (AD, FP, FG, CB). Disagreements between reviewers were resolved through discussion or with recourse to two arbitrators if required (SR, GS).

### Outcomes

The outcomes were the personality traits of the FFM: Neuroticism, Extraversion, Openness, Agreeableness, and Conscientiousness. When a study measured personality traits by questionnaires or inventories not specifically developed on the basis of the FFM (i.e., Brooks and McKinley Personality Inventory [19]), we decided to use the dimensions considered theoretically related to the FFM (e.g., neediness dimension reported in Henriques-Calado et al. [16] was considered as belonging to neuroticism, see Supplementary Table 1).

### Data extraction and coding

Data extracted and coded from the primary articles included: 1) characteristics of the publication: (e.g., authors, publication status, year of publication, journal); 2) characteristics of the sample (e.g., total sample size, gender was coded as the frequency of men in a sample; severity of dementia assessed by Clinical Dementia Rating Scale (CDR); and 3) measures assessing personality traits.

### Statistical analyses

We synthetized study data using meta-analytic methods. Initially, we computed the effect sizes from data reported in the articles (e.g., means and standard deviations; event or non event; p values) using Hedges’ g unbiased approach (like the Cohen d statistic). Negative values of the Hedges’ g indicated that AD patients had lower scores than HC on each personality dimension. The conventions used to interpret Hedges’ g are similar to Cohen’s d. According to Cohen’s criteria [20], values <0.20 are considered small effects, values of about 0.50 moderate effects, and values of about 0.80 large effects. For each effect size, 95% confidence interval, variance, standard error and statistical significance were computed. Effect sizes were pooled across studies for obtaining an overall effect size with the inverse-variance method. The random-effects model was used as a conservative approach to account for different sources of variation among studies and to generalize the meta-analytic finding beyond the studies herewith included.

Heterogeneity among the studies was assessed using Q and I^2^ statistics index [21]. A significant Q value indicates a lack of homogeneity of findings among studies; the proportion of observed variance that reflects real differences in effect sizes was estimated by I^2^. A value of 25, 50, and 75% was considered as low, moderate, and high, respectively [22]. Moreover, we conducted sensitivity analyses to check the stability of study findings, computing how the overall effect size would change removing one study at a time.

To further explain heterogeneity across study findings, we conducted moderator analyses with 5 moderators (i.e., age at evaluation, gender, years of schooling, severity of dementia assessed by the CDR, measures assessing personality traits), which were assessed by meta-regressions. Finally, publication bias analysis was performed to control that published studies could have a larger mean effect size than unpublished studies [23]. To explore the publication bias we applied the funnel plot, that is a scatter plot of the effects sizes estimated from individual studies against a measure of their precision (e.g., their standard errors). To evaluate the funnel plot more reliably, we employed the Egger’s regression method [24], which test the asymmetry of the funnel plot, with nonsignificant results indicative of absence of publication bias. Moreover, we applied the trim and fill procedure, an iterative non-parametric statistical technique, which evaluates the effect of potential data censoring on the result of the meta-analyses [25]. In this method, the absence of publication bias is indicated by zero trimmed studies, or in the presence of trimmed studies, by a trivial difference between the observed and the estimated effect sizes [26]. Statistical analyses were conducted with the meta-analytic software ProMeta 3.0.

## RESULTS

### Literature search

**Fig. 1:**
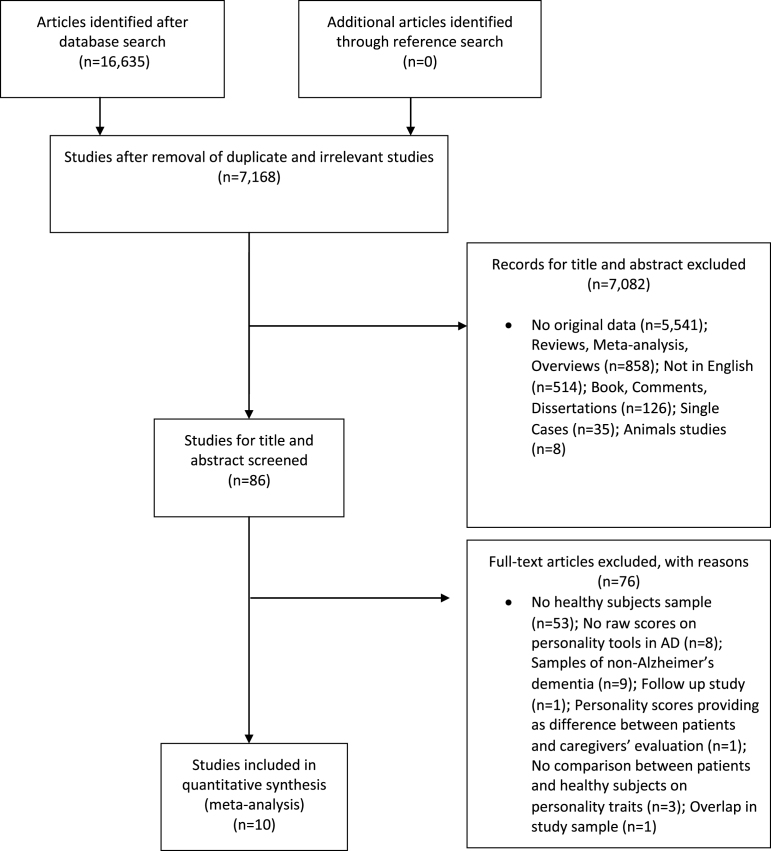
Flowchart of the selection process of primary studies.

Figure 1 showed the flow diagram based on PRISMA statement. The initial search identified 16,633 articles; after the removal of duplicates, we obtained 7,168 articles. Out of these, 7,082 articles were excluded based on their title and abstract. After full-text assessment, 86 studies were considered eligible. However, taking into account the inclusion and exclusion criteria, we excluded 76 articles. The reasons for their exclusion from the meta-analysis were reported in Fig. 1.

### Descriptive characteristics of studies included in the meta-analysis

The present meta-analysis is based on data extracted from 10 studies published from 1987 to 2017, which matched the eligibility criteria. Table 1 showed the characteristics of the 10 primary studies on personality traits in AD and HC groups and the personality tools used in each primary study to evaluate personality dimensions [3, 11–16, 27–29]. The AD sample included 603 patients with AD, with a mean age ranging from 66.6 to 81.3 years old, with mean years of schooling ranging from 7.61 to 15.8 years (in 5 studies the data of education were not reported). The HC sample included 679 subjects, with a mean age ranging from 52.2 to 75.8, with mean years of schooling ranging from 8.94 to 17 years (in 5 studies the data of the education were not reported). Moreover, none of the HCs was AD patients’ caregiver. In 7/10 primary studies, HC samples were selected from the community, whereas in the remaining 3 studies the information was not reported. The personality traits were assessed by both self-rated and informant-rated measures in 5/10 studies, whereas they were evaluated by informant-rated measures alone in 5/10 studies.

**Table 1:** Characteristics of primary studies included in the meta-analysis

Primary studies Authors	Country	AD patients (*n* = 603)							Healthy subjects (*n* = 679)				Type of sample	Diagnostic criteria	Self- or Informant- rating	Tools assessing personality traits	Personality dimensions
		*n*	Age (y)	Education (y)	Males (n)	Age at onset	Duration of AD	CDR	*n*	Age (y)	Education (y)	Males (n)					
Duchek et al. [11]	USA (Washington)	74	75.2 (9.3)	14.3 (3.1)	NR	NR	NR	0.5	36^*^	52.2 (4.8)	15.1 (2.8)	NR	Individuals from the community	CDR	Self- and Informant-ratings	NEO- FFI	Neuroticism, Extraversion, Openness, Agreeableness, Conscientiousness
		46	77.9 (8.9)	14.1 (3.2)	NR	NR	NR	1	131^**^	75.1 (10.2)	14.9 (3.9)	NR					
Henriques-Calado et al. [16]	Portugal (Lisbon)	44	81.3	7.61	0	NR	NR	NR	80	75.8	8.9	0	Individuals from the community	ICD, NINCDS-ADRDA	Self- and Informant-ratings	DEQ	Self-criticism, Dependency, Neediness, Connectedness
Petry et al. [3]	USA (Los Angeles)	30	72 (9.7)	NR	NR	NR	NR	NR	30	NR	NR	NR	NR	DSM- III	Informant-rating (pre and post diagnosis)	Brooks and McKinlay PI	Self-reliance, Down to earth, Maturity, Enthusiastic, Stable, Energetic, Reasonable, Happy, Easygoing, Affectionate, Kindness, Calm, Talkative, Even-tempered, Generous, Fond of company, Cautious, Sensitive
Pocnet et al [12]	Switzerland (Lausanne)	54	76.9 (8.5)	NR	15	NR	NR	NR	64	69.3 (8.7)	NR	29	Individuals from the community	NINCDS-ADRDA	Self- and informant-ratings	FFM and NEO-PI-R	Neuroticism, Extraversion, Openness, Agreeableness, Conscientiousness
Pocnet et al. [13]	Switzerland (Lausanne)	54	76.9 (8.5)	NR	15	NR	NR	NR	64	69.3 (8.7)	NR	29	Individuals from the community	NINCDS-ADRDA	Informant-rating (pre and post diagnosis)	NEO-PI-R	Neuroticism, Extraversion, Openness, Agreeableness, Conscientiousness
Sollberger et al. [29]	USA (San Francisco)	64	66.6 (11.7)	15.8 (3.3)	33	NR	NR	1 (0.5)	43	67.6 (9)	17 (2.7)	13	Individuals from the community	NINCDS-ADRDA, CDR, MMSE	Informant-rating	IAS	Assured/dominant, Arrogant/calculating, Cold hearted, Aloof/introverted, Unassured/submissive, Unassuming/ingenuous, Warm/agreeable, Gregarious/extraverted
Cummings et al. [27]	USA (Los Angeles)	30	71.2 (8.9)	NR	30	NR	6.6 (3.9)	NR	30	71.3 (5.7)	NR	30	NR	DSM-III-R, MMSE	Informant-rating	Brooks and McKinlay PI	Self-reliance, Down to earth, Maturity, Enthusiastic, Stable, Energetic, Reasonable, Happy, Easygoing, Affectionate, Kindness, Calm, Talkative, Even-tempered, Generous, Fond of company, Cautious, Sensitive
Roy et al. [28]	USA (New York)	119	75.0 (9.2)	13.8 (2.6)	50	NR	NR	NR	63	67.6 (6.0)	16.1 (2.5)	18	Individuals from the community	NINCDS-ADRDA	Self- and Informant-ratings	NEO- FFI	Neuroticism, Extraversion, Openness, Agreeableness, Conscientiousness
Rubin et al. [14]	USA (Washington)	44	71.4 (5)	NR	21	NR	NR	NR	58	71.7 (4.9)	NR	28	NR	CDR	Informant-rating	BDS	Passive behaviors, Agitated behaviors, Self- Centered
Henriques-Calado et al. [15]	Portugal (Lisbon)	44	81.36	7.61	0	NR	NR	NR	80	75.84	8.94	0	Individuals from the community	ICD, NINCDS-ADRDA	Self- and Informant-ratings	NEO-FFI	Neuroticism, Extraversion, Openness, Agreeableness, Conscientiousness

^*^middle age; ^**^older age; AD, Alzheimer’s disease; n, number; y, year; NR, not reported; CDR, Clinical Dementia Rating Scale; ICD, International Classification of Disease; MMSE, Mini-Mental State Examination; BDS, Blessed Dementia scale; DEQ, Depressive Experiences Questionnaire; IAS, Interpersonal Adjectives Scale; NEO-FFI, NEO Five-Factor Inventory; PI, Personality Inventory; NEO-PI-R, Revised NEO Personality Inventory; FFM, Structured interview for the Five-Factor Model.

### Meta-analytic results

We conducted 10 meta-analyses examining the comparison between AD patients and HC on the following personality traits (i.e., Neuroticism, Extraversion, Openness to experience, Agreeableness, and Conscientiousness). Out of these, 5 meta-analysis included only primary studies where personality traits were evaluated by informant-rated measures alone and 5 meta-analysis included primary studies where personality was explored by self-rated measures alone.

### Neuroticism

**Fig. 2:**
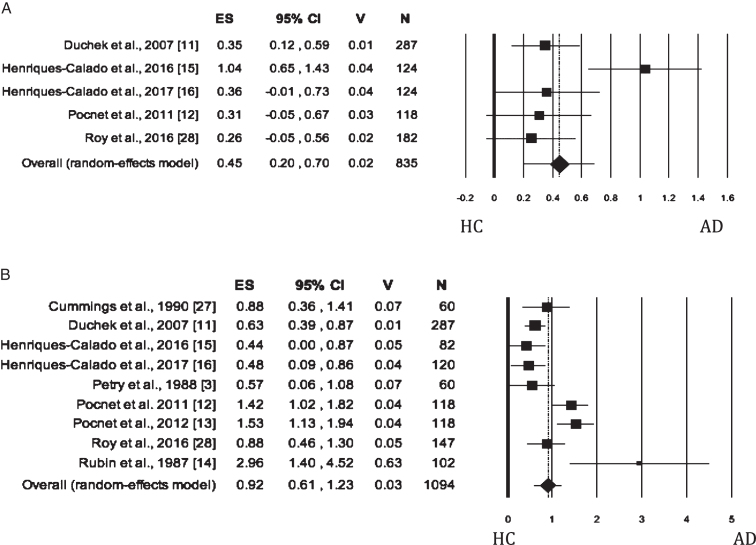
Forest plot for Neuroticism evaluated by self-rated (A) measures and informant-rated (B) measures, displaying effect size (Hedges’ g) calculated using a random effects model. ES, effect size; CI, confidence intervals; V, variance; N, total number of participants; AD, Alzheimer’s disease; HC, healthy subjects.

When Neuroticism was evaluated by either self-rated or informant-rated measures, the AD patients scored higher on Neuroticism than HC (self-rated measures: Fig. 2A; informant-rated measures: Fig. 2B). There was no publication bias, but the heterogeneity among the studies was high (Table 2). As for meta-analysis on informant-rated measures, the sensitivity analysis showed that the removal of the study by Rubin et al. [14] slightly reduced the effect size (Table 2); however, the heterogeneity remained significant and high (I^2^ = 76.54%).

**Table 2:** Summary of meta-analytic results of the following personality domains: Neuroticism, Extraversion, Openness, Agreeableness, and Conscientiousness

Domains/Outcomes	K	N	AD patients	Healthy subjects	Pooled effect size Hedges’ *g* (*p* value)	(95% Confidence Intervals)		Homogeneity statistics			Egger’s *t* test for publication bias	Trim and fill
						LL	UL	Q (df)	*p*	I^2^		
**Neuroticism**												
*Informant-rated version*	9	1094	531	563	0.92 (< **0.001**)	0.61	1.23	37.08 (7)	<0.001	78.43	1.41 (*p* = 0.200)	0
Sensitivity analysis after removing Rubin et al. [14]	8	992	487	505	0.85 (< **0.001**)	0.56	1.14	29.90 (7)	<0.001	76.59	0.62 (*p* = 0.559)	0
*Self-rated version*	5	835	381	454	0.45 (< **0.001**)	0.20	0.70	11.70 (4)	0.020	65.81	0.90 (*p* = 0.435)	0
**Extraversion**												
*Informant-rated version*	8	979	511	468	–0.78 (**0.009**)	–1.36	–0.19	117.79 (7)	<0.001	94.06	0.00 (*p* = 0.998)	0
Sensitivity analysis after removing Pocnet et al. [13]	7	861	457	404	–0.56 (**0.034**)	–1.08	–0.04	70.973 (6)	<0.001	91.52	0.53 (*p* = 0.620)	0
Sensitivity analysis after removing Pocnet et al. [12]	7	861	457	404	–0.58 (**0.037**)	–1.13	–0.04	78.68 (6)	<0.001	92.37	0.39 (*p* = 0.712)	0
*Self-rated version*	4	711	337	374	–0.76 (**0.044**)	–1.49	–0.02	60.89 (3)	<0.001	95.07	–1.70 (*p* = 0.231)	1
**Openness**												
*Informant-rated version*	7	872	447	425	–1.11 (< **0.001**)	–1.46	–0.77	29.25 (6)	<0.001	79.48	–0.94 (*p* = 0.390)	0
Sensitivity analysis after removing Roy et al. [28]	6	725	328	397	–1.12 (< **0.001**)	–1.52	–0.71	29.16 (6)	<0.001	82.85	–0.83 (*p* = 0.451)
*Self-rated version*	4	711	337	374	–1.15 (< **0.001**)	–1.58	–0.72	19.25 (3)	<0.001	84.41	–0.28 (*p* = 0.329)	0
**Agreeableness**												
*Informant-rated version*	9	1081	555	526	–0.42 (< **0.001**)	–0.61	–0.24	14.88 (8)	0.062	46.23	–2.30 (*p* = 0.055)	0
Sensitivity analysis after removing Rubin et al. [14]	8	979	511	468	–0.37 (< **0.001**)	–0.51	–0.22	8.48 (7)	0.292	17.49	–1.13 (*p* = 0.301)
*Self-rated version*	4	711	337	374	–0.44 (0.079)	–0.94	0.05	29.38 (3)	<0.001	89.79	–0.70 (*p* = 0.558)	1
**Conscientiousness**												
*Informant-rated version*	7	872	447	425	–1.12 **(0.012)**	–2	–0.24	177.55(6)	<0.001	96.62	–0.03 (*p* = 0.979)	0
*Self-rated version*	4	711	337	374	–0.39 (0.110)	–0.87	0.09	27.29 (3)	<0.001	89.01	–0.20 (*p* = 0.857)	0

K, number of studies; AD, Alzheimer’s disease; N, total number of participants; LL, Lower Limit; UP, Upper Limit; Q and I^2^ indicate heterogeneity statistics; df, degrees of freedom. Statistically significant values are reported in bold.

### Extraversion

**Fig. 3:**
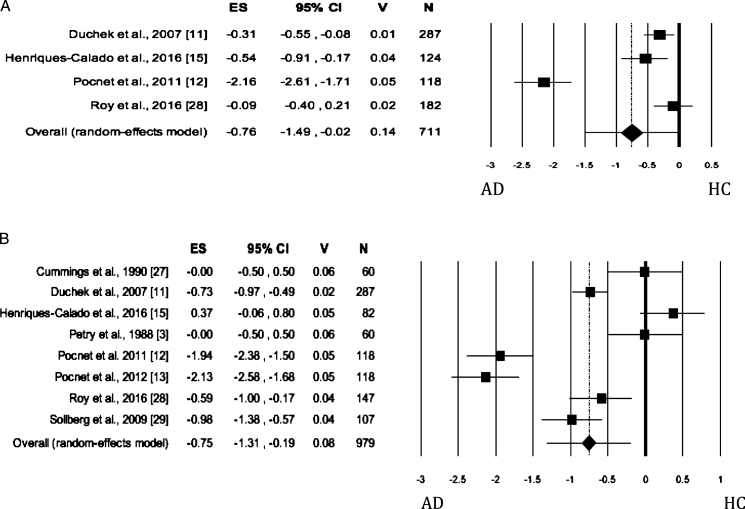
Forest plot for Extraversion evaluated by self-rated (A) measures and informant-rated (B) measures, displaying effect size (Hedges’ g) calculated using a random effects model. ES, effect size; CI, confidence Intervals; V, variance; N, total number of participants; AD, Alzheimer’s disease; HC, healthy subjects.

When Extraversion was evaluated by both self- and informant-rated questionnaires, the AD patients scored significantly lower than HC (Table 2; self-rated measures: Fig. 3A; informant-rated measures: Fig. 3B). There was no publication bias. The heterogeneity was high. As for informant-rated measures, the sensitivity analysis showed that the removal of Pocnet et al. [12] led to a slight reduction in effect size and in the level of the heterogeneity. We obtained similar results after removing Pocnet et al. [13].

### Openness

**Fig. 4:**
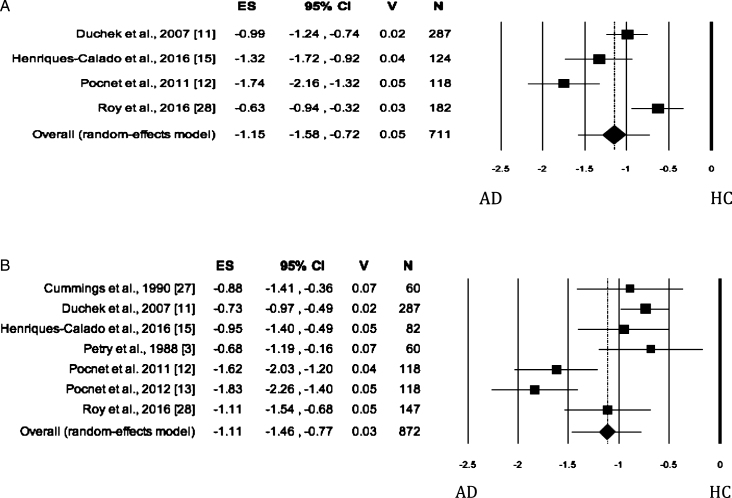
Forest plot for Openness evaluated by self-rated (A) measures and informant-rated (B) measures, displaying effect size (Hedges’ g) calculated using a random effects model. ES, effect size; CI, confidence intervals; V, variance; N, total number of participants; AD, Alzheimer’s disease; HC, healthy subjects.

When Openness was evaluated by both self- and informant-rated measures, the AD patients scored significantly lower than HC (self-rated measures: Fig. 4A; informant-rated measures: Fig. 4B) on this dimension. There was no publication bias, whereas the heterogeneity among studies was high (Table 2). As regards the Openness evaluated by informant-rated measures, we performed a sensitivity analysis revealing a slight increase in effect size after the removal of Roy et al.’s study [28] from the meta-analysis. However, the heterogeneity remained significant and high (I^2^ = 82.85%).

### Agreeableness

**Fig. 5:**
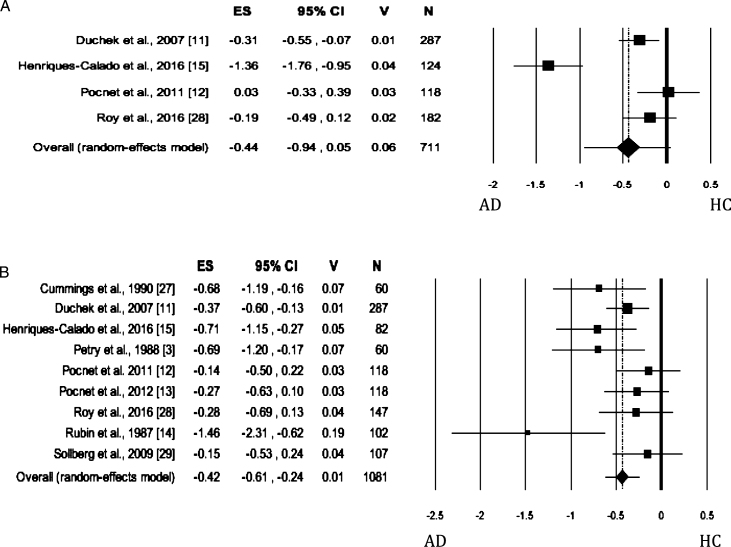
Forest plot for Agreeableness evaluated by self-rated (A) measures and informant-rated (B) measures, displaying effect size (Hedges’ g) calculated using a random effects model. ES, effect size; CI, confidence intervals; V, variance; N, total number of participants; AD, Alzheimer’s disease; HC, healthy subjects.

When the personality trait was evaluated by self-rated measures, no significant difference was found between AD and HC groups, with no publication bias (Table 2; self-rated measures: Fig. 5A). The Heterogeneity was high. When Agreeableness was evaluated by informant-rated measures, AD patients were perceived to be less agreeable than HCs (Table 2; informant-rated version: Fig. 5B). Neither publication bias nor heterogeneity across studies was significant.

### Conscientiousness

**Fig. 6:**
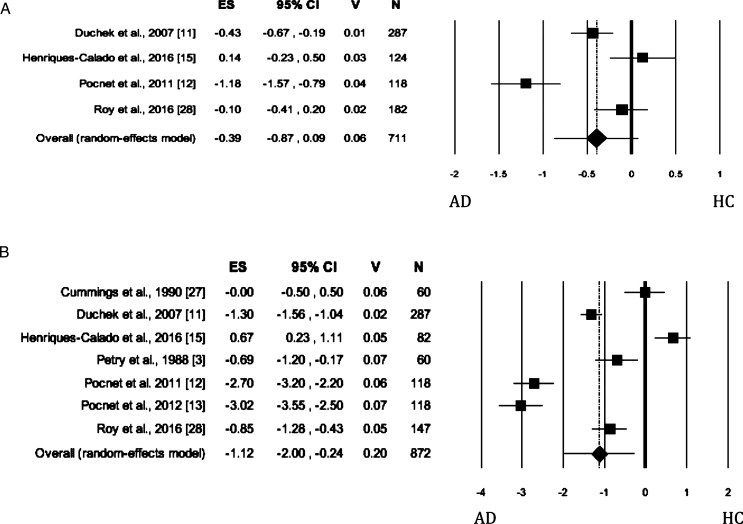
Forest plot for Conscientiousness evaluated by self-rated (A) measures and informant-rated (B) measures, displaying effect size (Hedges’ g) calculated using a random effects model. ES, effect size; CI, confidence intervals; V, variance; N, total number of participants; AD, Alzheimer’s disease; HC, healthy subjects.

When the trait was evaluated by self-rated measures, no significant difference was found between the AD and HC groups (Fig. 6A). The Egger’s test was significant and the heterogeneity was high. When Conscientiousness was evaluated by informant-rated measures, the AD patients showed lower scores than HC (Fig. 6B) on the dimension (Table 2). The Egger’s test was not significant, whereas the heterogeneity among the studies was high. Based on scanning of funnel plot and the sensitivity analysis, the removal of the study by Henriques-Calado et al. [13] from the meta-analysis led to a slight reduction in effect size (Effect size: –1.42, 95% Confidence Intervals, Lower Limit: –2.23, Upper Limit: –0.61; *p* = 0.001) and no publication bias. Instead, the heterogeneity remained significant but slightly reduced to 95.32%.

### Moderator analysis

Since few studies reported data on demographic aspects and type of tools used to assess the personality, we could not perform any meta-regression to evaluate their possible moderator effect on the relationship between AD and personality traits evaluated by means of self-rated measures.

When we considered only studies exploring personality traits by informant-rated measures, the meta-regressions revealed that age, frequency of men in the samples, and the type of tool used to evaluate personality did not reduce or increase the effect size of the relationship between each personality trait and AD. As for years of schooling, we found that this parameter moderated the relationship between agreeableness (B = 0.06, *p* = 0.029) or extraversion (B = –0.16, *p* = 0.012) and AD. However, according to Borenstein et al. [30], these results should be considered cautiously due to very few studies reporting the value of the years of schooling.

Since personality changes might be a function of AD, the disease duration from the diagnosis should be investigated as moderator of the effect size for each personality trait; however, we did not explore the issue as the disease duration was reported in only one study [27].

## DISCUSSION

The current meta-analysis revealed that personality profile of patients with a diagnosis of AD, when evaluated by informant-rated measures, was characterized by a high level of Neuroticism and a low level of Openness, Agreeableness, Conscientiousness, and Extraversion. Some of these results were confirmed even when personality traits were evaluated by self-rated questionnaires: in detail, significant differences between AD and HCs groups on Neuroticism, Openness, and Extraversion was confirmed. However, the discrepant results were detected on the Conscientiousness and Agreeableness domains: when the traits were evaluated by self-rated measures AD patients achieved similar scores to those of HCs on these two traits, whereas when the personality traits of AD patients and HCs was evaluated by their informants, AD patients were rated as having a lower level of Conscientiousness and Agreeableness than HCs. Potential demographic and clinical confounders did not influence the results.

Despite the high degree of the heterogeneity among the primary studies, our meta-analysis revealed a significant association between AD and a high level of Neuroticism both when evaluated by self-rated measures and when evaluated by informant-rated measures. Our finding was consistent with several behavioral previous studies [4, 11–16, 27, 28] and neuroimaging studies [31] revealing that Neuroticism was related to reduced volume in dorsomedial prefrontal cortex and hippocampus, with increased volume in the midcingulate cortex, all brain regions found to be altered by neurodegenerative processes of AD [32, 33]. Therefore, the significant association between Neuroticism and AD might reflect the fact that neurodegenerative processes early damage the same brain regions engaged in high level of Neuroticism. Moreover, our meta-analytic finding indirectly supports an association between this trait and worst performance on cognitive measures, particularly in memory tests (see meta-analysis of Luchetti et al. [9]) and the idea that Neuroticism is a greater risk factor for development of incident AD [4, 34].

In the current meta-analysis, despite high heterogeneity inter-study, we found that when AD patients and HCs’ personality was evaluated by their informant, AD patients showed lower scores than HCs on Conscientiousness and, therefore, they were described as less efficient, organized, goal-oriented, and more easy-going and disorderly, which may be considered another distinctive personality trait associated with AD. Similar to what was described for Neuroticism, low Conscientiousness is a significant predictor of conversion to dementia [4, 34] and has been associated with a worst cognitive status and a faster cognitive decline [3, 35]. Moreover, our finding of an association between low Conscientiousness and AD may be explained taking into account that low Conscientiousness was found to be associated with white matter lesions [36] and with reduced volume of lateral prefrontal cortex, a brain region engaged in planning and damaged in AD at different stages of disease [37]. Both high Neuroticism and low Conscientiousness are related to cigarette smoking [38], physical inactivity [39], obesity [40], and major depression, which in turn are risk factors for dementia [41]; on the basis of all above-mentioned assumptions and our finding, high Neuroticism and low Conscientiousness may be considered as two main distinctive personality traits of AD. It is noteworthy that when in the meta-analysis, we included only studies where AD patients and HCs self-reported their own personality traits, the relationship between low Conscientiousness and AD was not revealed. The absence of such difference might be secondary to unawareness of cognitive and behavioral disturbances, which occurs in AD patients. Moreover, this result might suggest that the source of inaccurate self-awareness in AD patients was that they failed to update their self-image [42]. Previous meta-analysis did not indicate the Extraversion as a risk factor for the development of cognitive decline over time [9, 10]. However, compared to healthy subjects, individuals with AD were described as characterized by decreased Extraversion after a diagnosis of AD [43]. This pattern has been reported in studies that measured the personality dimensions by self-reports [11, 33] or structured interviews to patients [12] and to caregivers [11]. Our results of a lower level of Extraversion trait in AD, obtained by both self-rated and informant-rated measures, were unexpected and might be considered as an indirect support to the idea that Extraversion (i.e., high Introversion) is a distinctive personality trait of AD patients which decrease over time; our results are in line with evidence that a low level of Extraversion can occur also in mild cognitive impairment (MCI) patients [44]. Moreover, the association between high Introversion and AD might be explained by the fact that amygdala and its connections with many cortical regions are the neural correlates of Introversion/Extraversion dimension and are reported as altered in MCI, a prodromal stage of dementia [45].

Since Openness to experiences refers to being interested in new people, places, and things and reflects the degree of intellectual curiosity, creativity, and preference for variety, high level of this personality trait seems to be a protective factor against cognitive decline and AD [46]. The relationship between Openness and cognitive decline may be explained by the cognitive reserve hypothesis [47, 48]. In fact, individuals with a high level of Openness are more frequently and intensively engaged in stimulating and cognitively-enriching activities in their lifetime and this engagement gives an advantage in cognitive functioning in later life (e.g., Chamorro-Premuzic and Furnham, [49]). Our results evidenced that a low level of Openness to experience (measured by self-rated and informant-rated measures) is a distinctive personality trait in AD patients as compared to HCs and might indirectly support the idea that a resilient personality profile which boosts subjects’ cognitive reserve may act as a protecting factor against cognitive decline in line with previous studies [49].

As for the Agreeableness, we did not find any significant difference between AD patients and HCs, when the personality trait was measured by self-rated measures. On the basis of our meta-analytic results, the Agreeableness dimension does not seem to be a distinctive personality trait associated with AD. Our results are consistent with case-control and longitudinal studies revealing no association between Agreeableness and dementia or MCI risk. However, when Agreeableness was evaluated by informant-rated measures, AD patients were perceived to be less agreeable than HCs. These findings are in line with those of Terracciano et al. [10] who found that agreeable subjects had a reduced risk of AD, although none of the studies included in their meta-analysis showed a significant association. The authors interpreted their results on the basis of the idea that individuals with low level of Agreeableness tend to be aggressive, antagonistic, and hostile and thus they are at high risk of cardiovascular diseases [50, 51] that, in turn, may contribute to increase the risk of AD. Similar to what has been abovementioned for Conscientiousness, the discrepancy between self-rated and informant-rated measures on the Agreeableness might reflect an inaccurate self-awareness of change of some specific personality traits in AD patients [42].

As for the strengths of the present study, we focused exclusively on the personality traits associated with AD including only results pertaining to clinically diagnosed AD and we included all five major dimensions of personality. However, a number of limitations of our meta-analysis needs to be taken into account. Firstly, personality has often been evaluated by different assessment tools that makes comparison difficult. In fact, we found high level of heterogeneity among the primary studies for each dimension of the personality. However, we tried to reduce this source of variation, choosing findings from those studies that employed personality measures linked to FFM. Moreover, we performed separate meta-analysis for each single personality trait by including AD patients’ personality results based exclusively either on self-rated measures or informant-rated measures; this methodological procedure allowed to reduce the level of heterogeneity across the primary studies. However, as for HCs’ personality, only some studies clearly specified that HCs’ personality traits were reported by their spouses or child [3, 15, 16]. By performing two separate meta-analysis for each personality trait, we obtained simultaneously both self-rated and informant-rated personality profile of AD patients compared to that of HCs and we revealed similarities or differences between self-rated and informant-rated personality profile of the two groups. It is noteworthy that the evaluation of AD patients’ personality profile by using both self-rated and informant-rated measures is relevant since patients with AD in advanced stages of the disease might be unable to provide information about their own personality profile. However, it has been evidenced that individuals with mild AD tended to describe their former personality [12] when asked to evaluate their current personality. This finding has been interpreted as a consequence of an inability to update their self-image [52] and thus on the basis of this latter consideration, it is likely that the personality profile revealed in our meta-analysis also reflects the premorbid personality specifically associated with AD. On the other hand, although the caregiver’s ratings of personality traits of AD patients seem to be more reliable than self-ratings, the high reliability of the caregiver’s ratings should be considered with caution. In fact, it is noteworthy that the use of family members to obtain personality data may lead to provide subjective judgments biased by the patients’ current symptoms such as apathy or depression [8].

Our meta-analysis included only cross-sectional studies and thus revealed cross-sectional differences between patients and controls. The cross-sectional nature of studies did not allow to elucidate the causal relationship between personality traits. On one hand, the association might indirectly support the idea of specific personality traits as risk factors for AD and on the other hand, it might suggest that some of the differences between AD and HC groups might reflect an adaptation process to the disease. In other terms, the diagnosis of AD may lead individuals to prefer familiar environments rather than novel ones, to be more detached from their family members; these behaviors would be reflected in lower level of Openness and higher level of Introversion. Further longitudinal studies should be performed to explore the causal relationship between personality traits and AD and should explore the change in personality from before to after diagnosis of AD.

The age of onset of AD, the disease duration, the clinical stage of the disease, and also prescribed drugs (e.g., sedative, anti-depressive, or behavior-modifying medication) might be possible factors influencing the degree of the relationship between personality traits and AD. These factors have been reported in a very few primary studies and therefore, their potential effect should be investigated in future studies.

In conclusion, the present meta-analysis revealed a distinctive personality profile in AD patients characterized by high levels of Neuroticism and low level of Extraversion and Openness to experiences; this profile is very similar to a premorbid personality found to be significantly associated with the development of AD over time. As for Conscientiousness and Agreeableness, no significant difference between AD and HC groups was revealed when AD patients and HCs self-reported their own personality traits, but AD patients were perceived as less agreeable and conscientious than HCs when AD patients’ personality was evaluated by an informant.

Our findings might have some clinical implications: in fact, they might support the idea that the evaluation of the personality profile has to be considered as part of prognostic models to identify individuals at greater risk of dementia. Our findings suggested that it should be relevant to evaluate five personality traits by using both self-rated and informant-rated measures to obtain a clear personality profile of AD patients. Moreover, the early evaluation of the personality in demented patients might provide interventions better matched to the individual’s personality in order to improve acceptability, adherence, and effectiveness of interventions [54]. Moreover, the early identification of the distinctive personality traits in AD patients might help clinicians to have good social interaction with the patients and to expect the occurrence of specific behavioral symptoms [55] in order to provide timely treatments. Since personality traits can influence the choice of the type of coping or interpretation of stressing situations, the identification of maladaptive personality traits (e.g., neuroticism) in AD might allow to detect those subjects who are at a greater risk for experiencing psychological distress and exploiting a maladaptive emotion-coping style such as avoidant coping or denial [56]. Thus, these patients could be addressed to psychoeducational interventions aiming at the development of problem-focused coping strategies [57]. Moreover, in the view of SAR [17], the profiling of AD patients’ personality can be useful to improve human-robot interaction and also to enhance the user satisfaction and robot acceptance since user profiling enables a robot to adapt its behavior with respect to his/her characteristics and preferences [58].

## Supplementary Material

Supplementary Material
