# Supplementary material for: Meta-Analysis of Personality Traits in Alzheimer’s Disease: A Comparison with Healthy Subjects
Source: J Alzheimers Dis. 2018 Feb 20;62(2):773–87. doi: 10.3233/JAD-170901 (PMC5842787; doi:10.3233/JAD-170901)
Supplement: Supplementary Material [file jad-62-jad170901-s001.docx]

**Supplementary Table 1.** Personality Dimensions considered theoretically related to the FFM and converted in outcomes of the meta-analysis (e.g., neediness dimension reported in Henriques-Calado et al. [1] was considered as belonging to neuroticism).

| **Primary studies** | **Test** | **Personality dimensions** | **Outcomes of the meta-analysis** |
| --- | --- | --- | --- |
| Duchek et al. [2] | NEO- FFI | Neuroticism | Neuroticism |
|  |  | Extraversion | Extraversion |
|  |  | Openness | Openness |
|  |  | Agreeableness | Agreeableness |
|  |  | Conscientiousness | Conscientiousness |
|  |  |  |  |
| Henriques Calado et al. [1] | DEQ | Neediness | Neuroticism |
|  |  |  |  |
| Petry et al. [3] | Brooks and McKinlay PI | Reasonable | Conscientiousness |
|  |  | Talkative | Extraversion |
|  |  | Happy | Neuroticism |
|  |  | Generous | Agreeableness |
|  |  | Down to earth | Openness |
|  |  |  |  |
| Pocnet et al. [4] | FFM | Neuroticism | Neuroticism |
|  |  | Extraversion | Extraversion |
|  |  | Openness | Openness |
|  |  | Agreeableness | Agreeableness |
|  |  | Conscientiousness | Conscientiousness |
| Pocnet et al. [5] | NEO-P-R | Neuroticism | Neuroticism |
|  |  | Extraversion | Extraversion |
|  |  | Openness | Openness |
|  |  | Conscientiousness | Conscientiousness |
|  |  |  |  |
| Sollberger et al. [6] | IAS | Warm/agreeable (LM) | Agreeableness |
|  |  | Gregarious/extraverted (NO) | Extraversion |
| Cummings et al. [7] | Brooks and McKinlay PI | Reasonable | Conscientiousness |
|  |  | Talkative | Extraversion |
|  |  | Down to earth | Openness |
|  |  | Happy | Neuroticism |
|  |  | Generous | Agreeableness |
|  |  |  |  |
| Roy et al. [8] | NEO- FFI | Neuroticism | Neuroticism |
|  |  | Extraversion | Extraversion |
|  |  | Openness | Openness |
|  |  | Agreeableness | Agreeableness |
|  |  | Conscientiousness | Conscientiousness |
|  |  |  |  |
| Rubin et al. [9] | DBS | Passive behaviors | Neuroticism |
|  |  | Self-Centered | Agreeableness |
|  |  |  |  |
| Henriques Calado et al. [10] | NEO- FFI | Neuroticism | Neuroticism |
|  |  | Extraversion | Extraversion |
|  |  | Agreeableness | Agreeableness |
|  |  | Openness | Openness |
|  |  | Conscientiousness | Conscientiousness |

BDS, Blessed Dementia scale; DEQ, Depressive Experiences Questionnaire; IAS, Interpersonal Adjectives Scale; NEO-FFI, NEO Five-Factor Inventory; PI, Personality Inventory; NEO-PI-R, Revised NEO Personality Inventory; FFM, Structured interview for the Five-Factor Model

**REFERENCES**

[1] Henriques-Calado J, Duarte-Silva ME, Sousa Ferreira A (2017) Anaclitic personality dimension in women with Alzheimer's disease: Comparison with control groups. *Pers Individ Dif* **109**, 166-171.

[2] Duchek JM, Balota DA, Storandt M, Larsen R (2007) The power of personality in discriminating between healthy aging and early-stage Alzheimer's disease. *J Gerontol B Psychol Sci Soc Sci* **62**, 353-361.

[3] Petry S, Cummings JL, Hill MA, Shapira J (1988) Personality alterations in dementia of the Alzheimer type. *Arch Neurol* **45**, 1187-1190.

[4] Pocnet C, Rossier J, Antonietti JP, von Gunten A (2011) Personality changes in patients with beginning Alzheimer disease. *Can J Psychiatry* **56**, 408-417.

[5] Pocnet C, Rossier J, Antonietti JP, von Gunten A (2012) Personality traits and behavioral and psychological symptoms in patients at an early stage of Alzheimer's disease. *Int J Geriatr Psychiatry* **8**, 276-283.

[6] Sollberger M, Stanley CM, Wilson SM, Gyurak A, Beckman V, Growdon M, Jang J, Weiner MW, Miller BL, Rankin KP (2009) Neural basis of interpersonal traits in neurodegenerative diseases. *Neuropsychologia* **47**, 2812-2827.

[7] Cummings JL, Petry S, Dian L, Shapira J, Hill MA (1990) Organic personality disorder in dementia syndromes: an inventory approach. *J Neuropsychiatry Clin Neurosci* **2**, 261-267.

[8] Roy S, Ficarro S, Duberstein P, Chapman BP, Dubovsky S, Paroski M, Szigeti K, Benedict RHB (2016) Executive function and personality predict instrumental activities of daily living in Alzheimer disease. *Am J Geriatr Psychiatry* **24**, 1074-1083.

[9] Rubin EH, Morris JC, Berg L (1987) The progression of personality changes in senile dementia of the Alzheimer's type*. J Am Geriatr Soc* **35**, 721-725.

[10] Henriques-Calado J, Duarte-Silva M, Ferreira AS (2016) Personality traits in women with Alzheimer’s disease: Comparisons with control groups with the NEO-FFI. *Pers Individ Dif* **101**, 341-347.
